# Draft genomes of *Methylobacteriaceae* isolated from sorghum grown in the arid environment of central Arizona

**DOI:** 10.1128/mra.00446-26

**Published:** 2026-07-23

**Authors:** Ivetka W. Noon, Marie Ojeh, Giovanni Melandri, Rebecca A. Schomer

**Affiliations:** 1 School of Plant Sciences, University of Arizona 124485, Tucson, Arizona, USA; California State University San Marcos, San Marcos, California, USA

**Keywords:** *Methylobacterium*, arid environment, plant-microbe interactions

## Abstract

Pink pigmented facultative methylotrophs can use one-carbon compounds as their sole carbon source and exist on leaf surfaces. Twelve isolates were collected from an arid agricultural environment in Arizona and cultured using a selective medium for methanol-utilizing bacteria. We present 12 draft genomes of multiple *Methylorubrum* species.

## ANNOUNCEMENT

*Methylobacteriaceae* are a family of pink pigmented methylotrophic Alphaproteobacteria that colonize the plant phyllosphere and rhizosphere, promoting plant growth through the production of phytohormones and nutrient bioavailability conversion (1). They can be found in aquatic, soil, and plant-associated environments (1). Their ability to metabolize one-carbon (C1) compounds makes them critical in carbon cycling, environmental sustainability, and developing biotechnology applications (2, 3).

To investigate genetic variability of methylotrophic bacteria isolated from an arid environment, we collected leaves of different sorghum accessions from a field trial conducted during the 2023 summer at the Maricopa Agricultural Center, Arizona (33°03′39.2″N, 111°58′40.7″W). Leaves were printed onto MP medium to enrich for methylotrophic growth: noble agar (1.5% w/v), 1× HEPES, and 0.1% v/v LaCl_3_, with 0.5% v/v methanol as the sole carbon source (4–6). Pure cultures were obtained by streaking pink pigmented colonies for isolation on the same medium 3–4 subsequent times and incubated at 28°C for 5–7 days.

Genomic DNA (gDNA) was collected for the isolates either using New England Biolabs Monarch Spin gDNA Extraction Kit (T3010L), followed by gel purification with the Zymoclean Gel DNA Recovery Kit (D4002) or Zymo Quick-DNA Fungal/Bacterial Miniprep (D6005) (Table 1). Bacterial genome sequencing was performed either by SeqCenter via Illumina short-read technology or by Plasmidsaurus using Oxford Nanopore Technology (ONT). Sequence technology and library preparation kits are indicated in Table 1. The raw reads generated from Illumina were further processed and analyzed using KBase (7). They were trimmed via Trimmomatic v0.36 with default parameters; quality was assessed with FastQC v0.12.1; and assembly was done using SPAdes v3.15.3 (7). CheckM v1.0.8 and Quast v4.4 were used to assess the genome quality before annotating with Prokka v1.14.5 (7). In parallel, raw reads generated from ONT with Dorado v4.3 Super-Accurate Basecalling software were processed by Filtlong v0.2.1 to remove the bottom 5% of reads, followed by assembly with Miniasm v0.3 to concatenate reads before using Flye v2.9.1 assembly (8–10). Medaka v1.8.0 was used to generate a consensus sequence before annotating with Bakta v1.11 (8, 11). Contig analysis was done with Bandage v0.8.1, and genome quality was checked with CheckM v1.2.2 and Quast v4.4 (7, 12, 13). Taxonomic classification for all samples was assigned using a combination of Fast ANI v.0.1.3 (Average Nucleotide Identity) and GTDB-Tk v1.7.0 against RefSeq genomes from National Center for Biotechnology Information (NCBI) (Table 1). These classifications were further refined using Mash v2.3 and Sourmash v4.6.2 against GenBank (7, 14–16).

**TABLE 1:** Metadata describing 12 *Methylobacteriaceae* draft genomes

Genome ID	Classification	Reference genome (RefSeq)	% ANI	% Complete	Genome size (Mb)	Contigs	% GC	Raw read count	Sequence technologies	Library preparation	Extraction kit	MCP signaling domain (PF00015)	SRA #	Accession #
IWN022	*Methylorubrum populi*	*Methylorubrum populi* BJ001, GCF_000019945.1	87.4	99.69	5.2	1	68.5	218,842	ONT PromethION, long read	Ultra-Long Sequencing Kit v14, Ligation Kit v14, and Assembly Kit v14	Zymo Quick-DNA Fungal/Bacterial Miniprep (D6005)	22	SRR34479654	GCA_977887345
IWN030	*Methylorubrum populi*	*Methylorubrum populi* BJ001, GCF_000019945.1	87.5	99.69	5.2	1	68.3	166,946	ONT PromethION, long read	Ultra-Long Sequencing Kit v14, Ligation Kit v14, and Assembly Kit v14	Zymo Quick-DNA Fungal/Bacterial Miniprep (D6005)	20	SRR34479652	GCA_977887495
IWN028	*Methylorubrum extorquens*	*Methylorubrum extorquens* AM1, GCF_000022685.1	96.5	98.96	6.1	4	67.7	49,322	ONT PromethION, long read	Ultra-Long Sequencing Kit v14, Ligation Kit v14, and Assembly Kit v14	New England Biolabs Monarch Spin gDNA Extraction (T3010L)	23	SRR34479653	GCA_977887135
IWN003	*Methylorubrum extorquens*	*Methylorubrum extorquens* AM1, GCF_000022685.1	96.6	99.27	5.7	126	68.0	2,307,512	Illumina NovaSeq X Plus, short reads	Illumina DNA Prep, (M) Tagmentation	New England Biolabs Monarch Spin gDNA Extraction (T3010L)	24	SRR34479655	GCA_977887115
IWN032	*Methylorubrum extorquens*	*Methylorubrum extorquens* AM1, GCF_000022685.1	96.7	99.37	5.7	1	68.0	149,577	ONT PromethION, long read	Ultra-Long Sequencing Kit v14, Ligation Kit v14, and Assembly Kit v14	Zymo Quick-DNA Fungal/Bacterial Miniprep (D6005)	23	SRR34479650	GCA_977887655
IWN014	*Methylorubrum extorquens*	*Methylorubrum extorquens* AM1, GCF_000022685.1	96.9	99.69	5.4	40	68.3	2,021,066	Illumina NovaSeq X Plus, short reads	Illumina DNA Prep, (M) Tagmentation	New England Biolabs Monarch Spin gDNA Extraction (T3010L)	30	SRR34479648	GCA_977887965
IWN011	*Methylorubrum aminovorans*	*Methylorubrum aminovorans* NBRC15686, GCF_022179725.1	97.41	99.69	5.1	1	68.6	73,305	ONT PromethION, long read	Ultra-Long Sequencing Kit v14, Ligation Kit v14, and Assembly Kit v14	Zymo Quick-DNA Fungal/Bacterial Miniprep (D6005)	27	SRR34479645	GCA_055524375.1
IWN039	*Methylorubrum aminovorans*	*Methylorubrum aminovorans* NBRC15686, GCF_022179725.1	97.44	99.69	5.2	1	68.6	137,367	ONT PromethION, long read	Ultra-Long Sequencing Kit v14, Ligation Kit v14, and Assembly Kit v14	Zymo Quick-DNA Fungal/Bacterial Miniprep (D6005)	19	SRR34479649	GCA_977887775
IWN044	*Methylorubrum aminovorans*	*Methylorubrum aminovorans* NBRC15686, GCF_022179725.1	97.45	99.69	5.3	49	68.4	1,760,969	Illumina NovaSeq X Plus, short reads	Illumina DNA Prep, (M) Tagmentation	New England Biolabs Monarch Spin gDNA Extraction (T3010L)	24	SRR34479646	GCA_977887125
IWN031	*Methylorubrum aminovorans*	*Methylorubrum aminovorans* NBRC15686, GCF_022179725.1	97.46	99.69	5.3	1	68.4	259,613	ONT PromethION, long read	Ultra-Long Sequencing Kit v14, Ligation Kit v14, and Assembly Kit v14	Zymo Quick-DNA Fungal/Bacterial Miniprep (D6005)	19	SRR34479651	GCA_977887635
IWN038	*Methylorubrum aminovorans*	*Methylorubrum aminovorans* NBRC15686, GCF_022179725.1	97.57	99.06	5.2	41	68.6	4,519,712	Illumina NovaSeq X Plus, short reads	Illumina DNA Prep, (M) Tagmentation	New England Biolabs Monarch Spin gDNA Extraction (T3010L)	30	SRR34479647	GCA_977887155
IWN021	*Methylorubrum thiocyanatum*	*Methylorubrum thiocyanatum* DSM 11,490*,* GCF_014138645.1	98.05	99.69	5.4	2	69.4	122,597	ONT PromethION, long read	Ultra-Long Sequencing Kit v14, Ligation Kit v14, and Assembly Kit v14	Zymo Quick-DNA Fungal/Bacterial Miniprep (D6005)	22	SRR34479644	GCA_977887145

Assemblies were generated for all 12 environmental isolates ranging from 1 to 126 contigs. Genome sizes ranged from 5.2 to 6.1 Mb with an average of 68.4 GC%. Genome information is provided in Table 1.

In KBase, core Clusters of Orthologous Groups genes were identified (7). Coding sequences in these genomes include genes involved in methanol and formaldehyde utilization as well as flagellar biosynthesis and chemotaxis. The 12 isolates each encoded between 19 and 30 methyl-accepting chemotaxis proteins (MCP), while 8 contained lanthanide-dependent dehydrogenases.

## Data Availability

This sequencing project has been deposited in NCBI. Raw reads are deposited under the BioProject accession number PRJNA1289777, and the assemblies are deposited under the BioProject accession number PRJEB98447. All data supporting this study have been deposited in FigShare (https://doi.org/10.6084/m9.figshare.30899027 and https://doi.org/10.6084/m9.figshare.31174207). Raw sequencing read and assembly accession numbers are listed in Table 1.
